# Effects of Interfacial Charge Depletion in Organic Thin-Film Transistors with Polymeric Dielectrics on Electrical Stability

**DOI:** 10.3390/ma3063614

**Published:** 2010-06-09

**Authors:** Jaehoon Park, Jin-Hyuk Bae, Won-Ho Kim, Min-Hoi Kim, Chang-Min Keum, Sin-Doo Lee, Jong Sun Choi

**Affiliations:** 1 School of Electrical Engineering, Seoul National University, Seoul 151-600, Korea; E-Mails: jaypark@snu.ac.kr (J.P.); bjh78@snu.ac.kr (J.-H.B.); wonho79@snu.ac.kr (W.-H.K.); mhkim80@snu.ac.kr (M.-H.K.); dlog2002@snu.ac.kr (C.-M.K.); 2 School of Electronic and Electrical Engineering, Hongik University, Seoul 121-791, Korea; E-Mail: jschoi@hongik.ac.kr (J.S.C.)

**Keywords:** organic thin-film transistors, stability, polymer, dielectrics, hydroxyl group

## Abstract

We investigated the electrical stabilities of two types of pentacene-based organic thin-film transistors (OTFTs) with two different polymeric dielectrics: polystyrene (PS) and poly(4-vinyl phenol) (PVP), in terms of the interfacial charge depletion. Under a short-term bias stress condition, the OTFT with the PVP layer showed a substantial increase in the drain current and a positive shift of the threshold voltage, while the PS layer case exhibited no change. Furthermore, a significant increase in the off-state current was observed in the OTFT with the PVP layer which has a hydroxyl group. In the presence of the interfacial hydroxyl group in PVP, the holes are not fully depleted during repetitive operation of the OTFT with the PVP layer and a large positive gate voltage in the off-state regime is needed to effectively refresh the electrical characteristics. It is suggested that the depletion-limited holes at the interface, *i.e.*, interfacial charge depletion, between the PVP layer and the pentacene layer play a critical role on the electrical stability during operation of the OTFT.

## 1. Introduction

Organic semiconductors are essential for fabricating active elements in flexible electronics. For example, organic thin-film transistors (OTFTs) have been extensively studied with a view toward promising applications in the fields of large-area displays, disposable chips, and various sensors [1,2,3,4] since the field-effect mobility in the OTFT is as large as 5 cm^2^/Vs which is comparable or even superior to that in an amorphous silicon TFT [5]. Besides the magnitude of the mobility, the reliability of the OTFT including the environmental and electrical stabilities is one of critical issues for the full-scale application in electronics. Recently, it was reported that the environmental stability of the OTFT is strongly influenced by oxygen and moisture in ambient air [6,7], and thus several passivation methods have been suggested to enhance the environmental stability [8,9,10]. With regard to electrical stability, the bias stress on the OTFT results in a large shift of the threshold voltage, thereby degrading the device performances. However, a more comprehensive picture of the electrical instability of the OTFT still remains to be obtained since it involves complicated phenomena of charge transport and material properties of the OTFT.

Considering that main advantages of the OTFTs are mechanical, flexibility and low-cost manufacture for large-area electronics, polymer materials such as poly(4-vinyl phenol), poly(vinyl alcohol), and poly(methyl methacrylate) are very useful for preparing gate dielectric layers of the OTFTs due to the solution processability [11,12,13,14,15]. The polymeric dielectrics, however, are known to be less reliable than inorganic dielectrics in the electrical performances of the OTFT under bias stress conditions. It was reported previously that three possible mechanisms are mainly responsible for the electrical instability of the OTFT [16]; charge injection from the gate electrode into the polymeric dielectric layer, the polarization effect of the polymeric dielectric layer, and trapped charges at the interface between polymeric dielectric and organic semiconductor layers. Among them, the effects of the polarization and trapped charges are directly related to the physic-chemical properties and surface morphologies of the polymer dielectrics. Therefore, it is important to examine the electrical stability of the OTFT by varying the interfacial nature of the polymeric dielectric. In addition to a long-term bias stress at a fixed gate voltage, a short-term bias stress during repetitive operation is needed to assess the electrical stability of the OTFT which determines the optimum driving voltage and switching speed in integrated circuits. Such short-term bias stress measurements provide information about the charge transport depending on the gate dielectric, particularly, charge accumulation and depletion processes.

In this work, we have investigated two types of the OTFTs consisting of an organic semiconductor of pentacene, in one of which a polymeric dielectric of poly(4-vinylphenol) (PVP) was used and in the other polystyrene (PS) was used, to understand the interfacial effect of the polymer dielectric on the electrical stability of the OTFTs. The electrical properties and the surface energies of the PVP and PS layers, determined using metal-insulator-semiconductor (MIS) structures and contact angles, were used to analyze the electrical instabilities of the OTFTs with two polymeric dielectrics. In contrast to the PS case, the OTFT with the PVP layer showed substantial variations in the threshold voltage, the on/off current ratio, and the field-effect mobility under short-term bias stress conditions. It is suggested that those variations are likely attributed to the depletion-limited holes that are not fully depleted in the presence of the hydroxyl group in the PVP layer during repetitive operation. A large positive bias voltage at the gate in the off-state regime was found to refresh the electrical characteristics of the OTFT with the PVP layer, implying that the interfacial interactions related to the hydroxyl group in the PVP layer can be balanced by the gate bias voltage.

## 2. Results and Discussion

### 2.1. Characteristics of PVP and PS Layers

**Figure 1:**
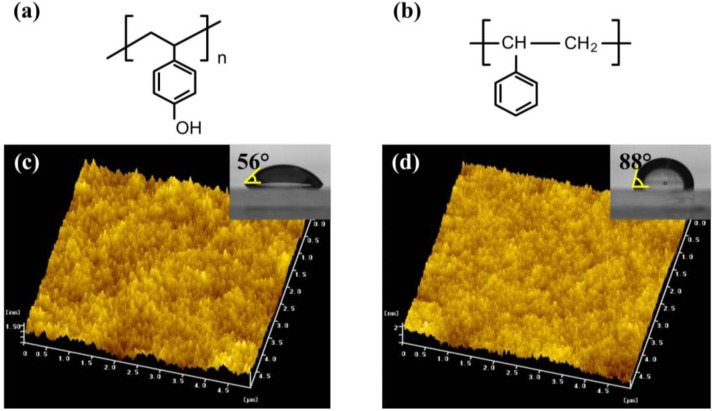
Chemical structures of **(a)** PVP and **(b)** PS. AFM images (5 μm × 5 μm) of **(c)** the PVP and **(d)** PS layers. The insets show the contact angles on both polymer layers.

We first examine the surface energy and the morphological roughness of the PVP layer and that of the PS layer. As shown by the chemical structures of PVP and PS in Figure 1(a) and Figure 1(b), respectively, the hydroxyl group (−OH) exists only in the PVP layer. Distilled-water contact angle measurements were carried out to investigate the surface energies of the two dielectric layers of PVP and PS. The contact angle of a sessile drop on each dielectric layer was directly measured by aligning a tangent with the drop profile at the point of contact with the surface. From insets of Figure 1(c) and Figure 1(d), it was found that the contact angle on the PVP layer (*θ_0_* ≈ 56°) is much smaller than that on the PS layer (*θ_0_* ≈ 88°). The surface free energy (*γ_P_*) of each layer can be calculated using the following equation [17]:

(1) $$\gamma_{p}=\frac{\gamma_{W}}{4}\times {\left(1+\text{cos }\theta_{0}\right)}^{2}$$

where *θ_0_* is the contact angle at equilibrium and *γ_W_* is the water surface free energy (73 mJ/m^2^). The values of *γ_P_* of the PVP and PS layers were determined to be about 44.3 and 19.5 mJ/m^2^ from Equation 1, respectively. This indicates that the surface of the PVP layer containing the hydroxyl group is relatively hydrophilic and polar in comparison to that of the PS layer. From the atomic force microscope (AFM) images in Figure 1(c) and Figure 1(d), the root-mean-square roughness values are measured to be about 2.1 and 2.3 nm for the PVP and PS layers, respectively. Note that there was no considerable difference in the surface morphology between the two layers. Therefore, the electrical stability of the OTFT will be examined in terms of the effect of the hydroxyl group only.

### 2.2. Pentacene Films Grown on PVP and PS Layers

**Figure 2:**
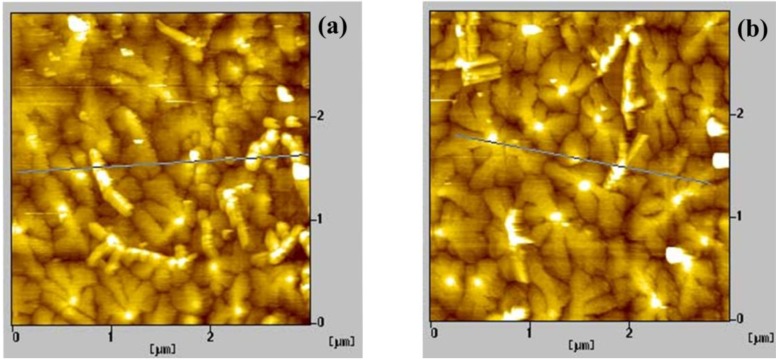
AFM images (3 μm × 3 μm) of the pentacene films grown on **(a)** the PVP and **(b)** PS layers.

In general, the grain size of pentacene molecules on a dielectric layer is known to strongly depend on the surface energy and the deposition rate [18]. For comparing the electrical stabilities of the OTFTs on two different dielectric layers, it is important to produce similar grain sizes in the pentacene films. In our study, the deposition rate was varied as described in the experimental section. For the PVP case, a faster deposition rate was used than the PS case due to the larger surface energy. In the pentacene films on both PVP and PS layers, the surface roughness was about 2.1 nm. Figure 2(a) and Figure 2(b) show the AFM images of the 60-nm-thick pentacene films deposited onto the PVP and PS layers, respectively.

### 2.3. Electrical Characteristics of OTFTs with Different Dielectric Layers

**Figure 3:**
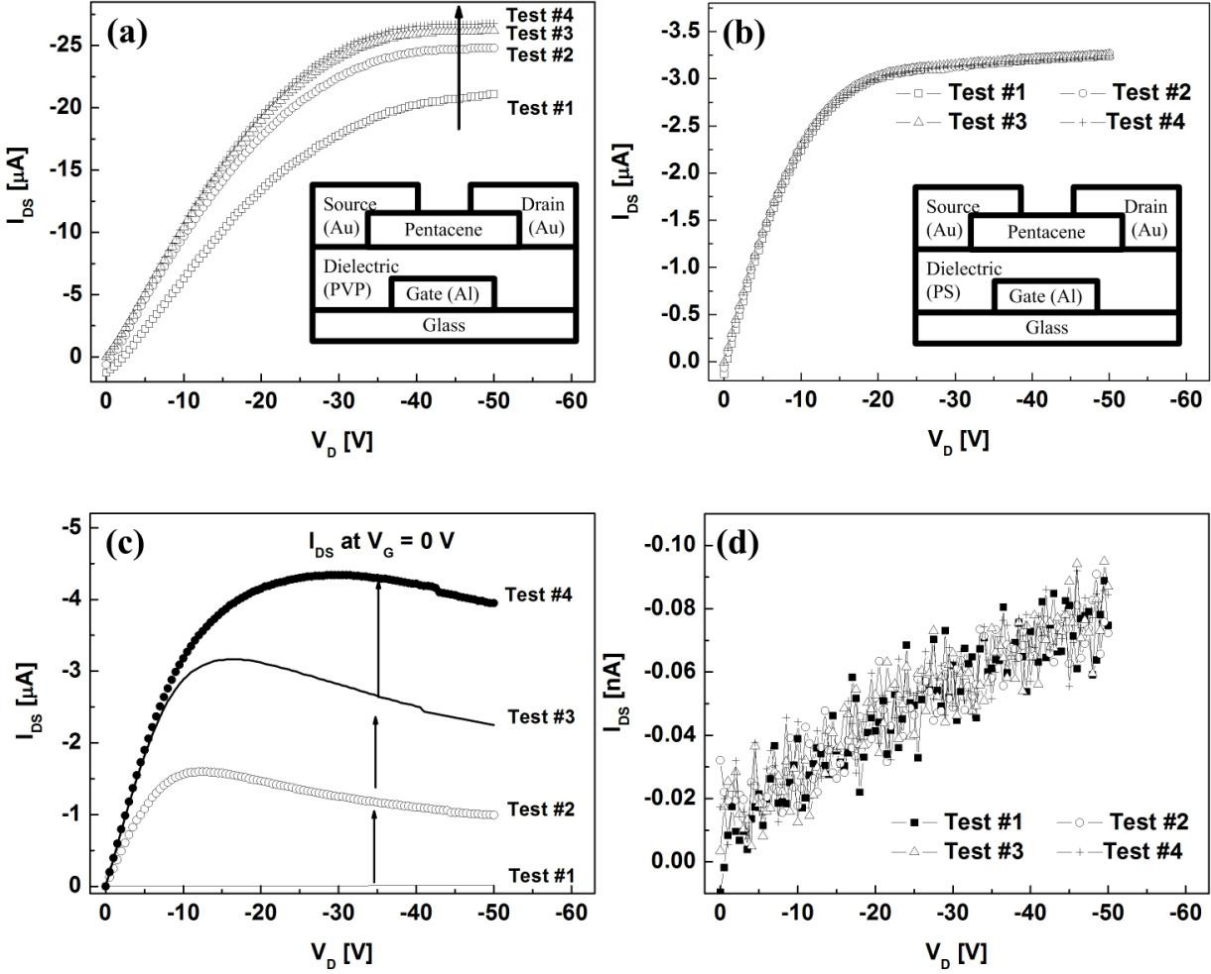
Output characteristics of the OTFTs with **(a)** the PVP layer and **(b)** the PS layer at *V_G_* = −30 V in sequence. The insets show the schematic representations of the fabricated OTFTs. Output characteristics of the OTFTs with **(c)** the PVP layer and **(d)** the PS layer at *V_G_* = 0 V during repetitive measurements.

Typical transistor characteristics were analyzed in a dark box under ambient air. In order to measure the output characteristics, the drain-source voltage (*V_D_*) was varied from 0 to −50 V at a sweep step of −0.5 V at two gate-source voltages (*V_G_*) of 0 and −30 V in sequence. It was repetitively carried out four times to investigate the electrical stabilities of the fabricated OTFTs under short-term bias stress conditions. Note that the short-term bias stress on the device can be effectively examined under such repetitive operation. The drain current (*I_DS_*) obtained at the *n*^th^ sequence is denoted as ‘Test #*n*’ in Figure 3(a) and Figure 3(b). It is observed that for the PVP case, *I_DS_* substantially increased during repetitive operation while for the PS case, it was negligible. Moreover, for the OTFT with the PVP layer, *I_DS_* values even at *V_G_* = 0 V kept increasing under repetitive operation as shown in Figure 3 (c). However, it is clear from Figure 3(d) that *I_DS_* remained essentially the same.

**Figure 4:**
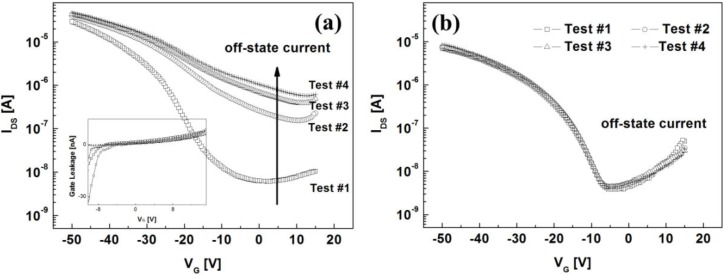
Transfer characteristics of the OTFTs with **(a)** the PVP and **(b)** PS layers at *V_D_* = −30 V according to the measurement sequence. The inset of Figure 4(a) shows the gate-leakage currents in the off-state regime.

The transfer characteristics of two types of the OTFTs at *V_D_* = −30 V are shown in Figure 4. The gate voltage of *V_G_* was varied from 15 to −50 V at a sweep step of −0.5 V. Important device parameters are summarized in Table 1. For the OTFT with the PVP layer, the off-state current rapidly increased during repetitive operation, thereby degrading the on/off behavior while the gate-leakage current in the off-state regime was nearly unchanged as shown in Figure 4 (a). Furthermore, the threshold voltage (*V_T_*) significantly shifted toward the positive direction and the field-effect mobility decreased by about 40%. Such performance degradation under a short-term bias stress condition limits fast-switching and high-speed operation of the OTFT. In contrast to the PVP case, the electrical characteristics of the OTFT with the PS layer remained constant as shown in Figure 4(b). The slight increase in the field-effect mobility might be associated with defect states between the PS layer and the pentacene layer during repetitive operation.

The increase in *I_DS_* for the OTFT with the PVP layer suggests that holes are not fully depleted in the presence of the hydroxyl group in the PVP layer during repetitive operation. It is physically reasonable that those depletion-limited holes would remain at the interface between the PVP layer and the pentacene layer under short-term bias stress conditions, so they contributed to the *I_DS_* conduction through consecutive measurements and consequently deteriorated the electrical stability of the OTFT.

**Table 1:** Characteristic parameters of the OTFTs with the PVP and PS layers according to the measurement sequence.

Dielectric Layer		Threshold Voltage [V]	On/Off Current Ratio	Field-Effect Mobility [cm^2^/Vs]
PVP	Test #1	−22.6	4.1 × 10^3^	0.36
	Test #2	−11.5	3.2 × 10^2^	0.25
	Test #3	−14.6	1.4 × 10^2^	0.22
	Test #4	−5.3	8.7 × 10^1^	0.21
PS	Test #1	−12.3	1.8 × 10^3^	0.82
	Test #2	−12.1	1.7 × 10^3^	0.88
	Test #3	−12.1	1.8 × 10^3^	0.89
	Test #4	−12.3	1.9 × 10^3^	0.98

**Figure 5:**
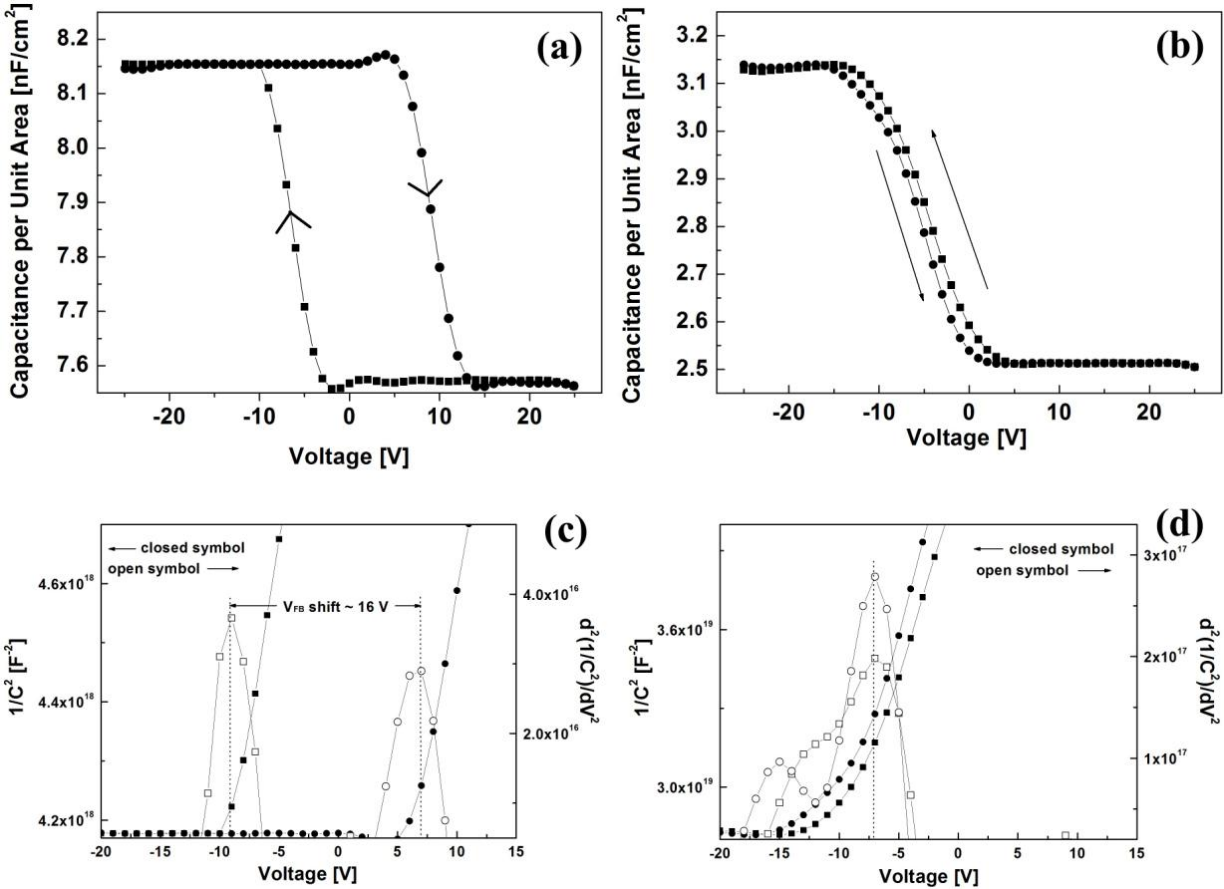
*C*-*V* curves for the MIS capacitors with **(a)** the PVP and **(b)** PS layers. Plots of 1/*C*^2^ and *d*^2^(1/*C*^2^)/*V*^2^ *versus* *V* for the capacitors with **(c)** the PVP and **(d)** PS layers.

Figure 5(a) and Figure 5(b) show the capacitance-voltage (*C*-*V*) characteristics of the MIS capacitors with the PVP and PS layers. The measurements were carried out using a small ac signal with the amplitude of 10 mV at 100 kHz. From the plots of 1/*C*^2^ *versus* *V* [19], the flat-band voltages (*V_FB_*) of the fabricated capacitors were determined as shown in Figure 5(c) and Figure 5(d). By applying the voltage successively from a positive-to-negative scan to a negative-to-positive scan, the MIS capacitor with the PVP layer exhibits a large hysteresis in the *C*-*V* curve with the positive *V_FB_* shift of 16 V upon the voltage sweep direction while the MIS capacitor with the PS layer shows only a slight hysteresis with no *V_FB_* shift. This clockwise hysteresis has been reported for the OTFTs composed of gate dielectrics containing polar functional groups and mobile ions [20,21]. Based on the energy band diagram model in the MIS capacitor, a positive *V_FB_* shift in the capacitor consisting of the *p*-type pentacene layer suggests the existence of depletion-limited holes at the PVP/pentacene interface and thus a larger positive voltage is required to fully deplete those holes. The density of interfacial holes present between the PVP layer and the pentacene layer can be estimated by the *V_FB_* shift using the following equation [22]:

(2) $$\Delta V_{FB}=\frac{\Delta Q_{\text{int}}}{C_{i}}$$

where *Q_int_* is the charge density at the interface between the gate dielectric and the semiconductor layer, and *C_i_* is the capacitance of a dielectric layer. From the measured value of *C_i_* ≈ 12.49 nF/cm^2^ for the PVP layer, the interfacial hole density was found to be about 1.2 × 10^12^ cm^−2^. This large hole density indicates that the depletion-limited holes at the interface between the PVP layer and the pentacene layer are dominantly involved in the electrical instability of the OTFT with the PVP layer during repetitive operation. The existence of the depletion-limited holes may result from the absorption of water by the hydrophilic PVP layer containing hydroxyl groups causing slow polarization [21]. Note that polar molecules such as water influence on the electrical characteristics of the OTFTs under a short-term bias stress condition.

### 2.4. Suppression of Electrical Instabilities of OTFT with PVP Dielectric Layer

**Figure 6:**
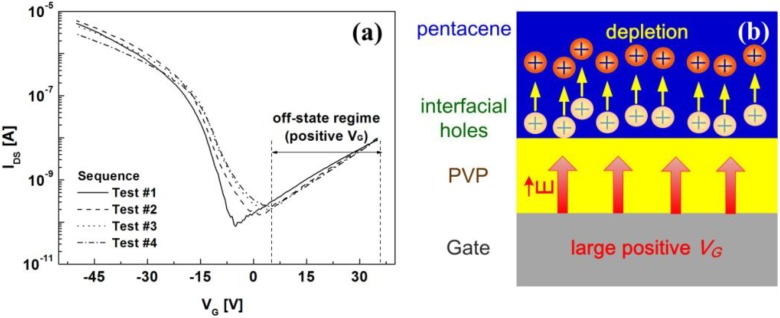
**(a)** Transfer characteristics of the OTFT with the PVP layer. **(b)** Schematic diagram of the depletion mechanism of interfacial holes by a large positive gate bias.

Let us now describe how the electrical instabilities of the OTFT with the PVP layer can be suppressed by a large positive gate voltage in the off-state regime. Since the depletion-limited holes are likely attributed to the hydroxyl group in the PVP layer, a hydroxyl-group-free surface is desirable for the stability of the OTFT. Although the cross-linking process of PVP, or the surface treatment with a self-assembled monolayer (SAM), is expected to improve the electrical stability of the OTFT with the PVP layer, hydroxyl groups on the PVP layer are not fully eliminated. A relatively large positive gate bias in the off-state regime is an alternative for stable operation of the OTFT with the PVP layer. The transfer characteristics were measured using *V_G_* varied from 35 to −50 V at a sweep step of −0.5 V. Figure 6(a) clearly shows the electrical stability the OTFT with the PVP layer during repetitive operation. The mechanism can be described in terms of the depletion of interfacial holes under the influence of a large positive gate voltage. Figure 6(b) shows that a large positive gate voltage in the off-state regime produces an electric field to deplete interfacial holes present between the PVP layer and the pentacene layer, and thus it effectively refreshes the electrical characteristics of the OTFT. In other words, the interfacial interactions related to the hydroxyl group in the PVP layer can be simply balanced by the gate bias voltage. For *n*-type OTFTs, a large negative gate voltage in the off-state regime may enhance the depletion of interfacial electrons.

## 3. Experimental Section

For the fabrication of the OTFT, a 150-nm-thick Al gate electrode was deposited on a glass substrate through a first shadow mask. The PVP (Sigma Aldrich, 6 wt % in ethanol) or PS (Sigma Aldrich, 2 wt % in chloroform) layer was spin-coated on top of the gate electrode. The PVP layer was baked at 110 °C for 60 min in a dry oven, followed by precuring at 60 °C for 10 min. The PS layer was baked at 100 °C for 60 min in a dry oven, followed by at 80 °C for 10 min. The thicknesses of the two polymeric dielectrics were about 400 nm. A 60-nm-thick pentacene layer was thermally deposited onto each dielectric layer through a second shadow mask. The deposition rate of the pentacene layer was 0.5 nm/s for the PVP-coated substrate and 0.1 nm/s for the PS-coated substrate. Bottom-gate/top-contact OTFTs were constructed using 40-nm-thick Au source/drain electrodes where the channel length (*L*) and width (*W*) were 90 and 300 μm, respectively. All evaporation processes were carried out under a base pressure of about 1.6 × 10^−6^ Torr. In order to study the electrical characteristics at the interface between each polymeric dielectric layer and the pentacene layer, the MIS capacitor was fabricated through the identical processes.

The surface energy of each polymeric dielectric was evaluated from the contact angle measured using a contact angle meter GSA10 (KRUSS Co. Ltd.). The surface morphologies of the dielectric layer and the pentacene film on each dielectric layer were exmained by AFM (XE150, PSIA Inc.). The current-voltage and capacitance-voltage characteristics of the fabricated devices were measured with a semiconductor parameter analyzer (EL 421C, Elecs Co.) and an impedance analyzer (HP 4192A, Agilent Technologies), respectively.

## 4. Conclusions

We present results for the electrical characteristics of two types of the pentacene-based OTFTs with two different polymeric dielectrics, PS and PVP, to understand the interfacial effect of the polymer dielectric on the electrical stability of the OTFTs. The OTFT with the PVP layer showed a substantial increase in the drain current and a positive shift of the threshold voltage under short-term bias stress conditions while the PS layer case exhibited no change. Moreover, a significant increase in the off-state current was observed in the OTFT with the PVP layer. It is suggested that such variations are likely attributed to the depletion-limited holes that are not fully depleted in the presence of the hydroxyl group in the PVP layer during repetitive operation. This is supported by the positive shift of the flat-band voltage in the *C-V* plots in the PVP case. A large positive gate voltage in the off-state regime effectively refreshed the electrical characteristics of the OTFT with the PVP layer by balancing the interfacial interactions related to the hydroxyl group in the PVP layer. Interfacial charge depletion between the PVP layer and the pentacene layer is found to play a critical role on the electrical stability during repetitive operation of the OTFT.
